# Prognostic impact of KRAS mutant type and MET amplification in metastatic and recurrent gastric cancer patients treated with first-line S-1 plus cisplatin chemotherapy

**DOI:** 10.18632/genesandcancer.96

**Published:** 2016-01

**Authors:** Satoshi Matsusaka, Takashi Kobunai, Noriko Yamamoto, Keisho Chin, Mariko Ogura, Gotaro Tanaka, Kazuaki Matsuoka, Yuichi Ishikawa, Nobuyuki Mizunuma, Toshiharu Yamaguchi

**Affiliations:** ^1^ Department of Gastroenterology, Cancer Institute Hospital of the Japanese Foundation for Cancer Research, Tokyo, Japan; ^2^ Translational Research Laboratory, Taiho Pharmaceutical Co., Ltd., Tokyo, Japan; ^3^ Department of Pathology, Japanese Foundation for Cancer Research, Tokyo, Japan; ^4^ Applied Pharmacology Laboratory, Taiho Pharmaceutical Co., Ltd., Tokushima, Japan; ^5^ Department of Gastroenterological Surgery, Cancer Institute Hospital of the Japanese Foundation for Cancer Research, Tokyo, Japan

**Keywords:** gastric cancer, KRAS, MET, DNA copy number, immunohistochemistry

## Abstract

Receptor tyrosine kinase (RTK)-related genes, including *HER2, EGFR, MET*, *FGFR2* and *KRAS*, are target molecules that are clinically beneficial in gastric cancer (GC). We investigated the correlation between RTK-related genes and the curative effect of first-line S-1 plus cisplatin (SP) combination chemotherapy in metastatic and recurrent GC. We enrolled 150 patients with histopathologically confirmed metastatic and recurrent GC treated with SP. *KRAS* mutation was detected using direct sequencing. DNA copy number was measured by real-time PCR. Formalin-fixed paraffin-embedded specimens were examined immunohistochemically for HER2, EGFR, FGFR2 and MET. Among 144 patients, *KRAS* mutation was detected in five (3.5%) at codon 12 and one (0.7%) at codon 13. *FGFR2*, *EGFR*, *HER2*, *MET* and *KRAS* gene amplification was suggested in 4.4%, 5.9%, 9%, 3.7% and 10.3% of patients, respectively. *KRAS* mutation, but not *KRAS* amplification, was associated with significantly shorter overall and progression-free survival. MET membranous overexpression was associated with a significantly higher tumor response. *MET* amplification was associated with significantly shorter overall survival. We show for the first time that *KRAS* mutation and *MET* amplification are promising predictive markers in metastatic and recurrent GC patients treated with SP. *KRAS* status may be a useful prognostic marker in patients treated with SP.

## INTRODUCTION

5-fluorouracil (including oral fluoropyrimidines S1 or capecitabine) with a platinum analog (cisplatin or oxaliplatin) is the most widely accepted first-line chemotherapy regimen for metastatic gastric cancer (GC) in Japan and other countries. S1 plus cisplatin (SP) combination therapy was significantly superior to S1 alone as first-line treatment for metastatic and recurrent GC in the phase III SPIRITS trial [1]. Current chemotherapy has improved survival in GC patients, although the survival rate remains low [2]. New molecular-targeting agents are urgently needed to achieve clinical benefits for patients with GC and improve their survival.

Deng *et al*. [3] have reported that five genes, *HER2*, *EGFR*, *MET*, *FGFR2* and *KRAS*, which are associated with the receptor tyrosine kinase (RTK)/RAS pathway, amplify the DNA copy number in GC. New drugs have been developed that target the RTK/RAS pathway. Trastuzumab (Herceptin) is a monoclonal antibody that specifically targets HER2 by directly binding with its extracellular domain [4]. Based on the results of the ToGA study, trastuzumab has become the first antimolecular targeting drug for patients with HER2-positive advanced GC [5]. The epidermal growth factor receptor (EGFR) pathway plays a core role in regulating tumor cell growth and survival, and is associated with poor prognosis in GC [6]. The EXPAND [7] and REAL3 [8] trials are two concurrent randomized phase 3 trials assessing the addition of anti-EGFR monoclonal antibodies for first-line treatment of upper gastrointestinal cancer. However, these studies suggest that addition of EGFR antibodies does not convey additional benefit for patients with advanced gastric and gastroesophageal junction cancer. Hepatocyte growth factor (HGF) and its receptor MET promote the proliferation, migration and survival of tumors, and are associated with poor prognosis in GC [9-12]. Rilotumumab, which is a fully human IgG2 monoclonal antibody against HGF, showed anti-tumor activity in a phase Ib and II study [13]. A phase II study [RILOMET-1 (NCT01697072)] has confirmed these results in patients with MET-positive gastric and esophagogastric junction cancer [14]. Fibroblast growth factor receptor (FGFR)2 is an RTK that regulates cell growth and development. *FGFR2* amplification is associated with tumor cell proliferation, survival of GC cell lines [15], and indicates poor prognosis in patients with GC [16].

Few studies have examined the effect of RTK/RAS-related genes on clinical outcomes in GC. The aim of this study was to investigate the correlation between RTK/RAS-related genes and the curative effect of SP combination chemotherapy in metastatic and recurrent GC.

## RESULTS

### Patients

We enrolled 150 patients with metastatic and recurrent GC treated with first-line SP combination chemotherapy. Their baseline characteristics are summarized in Table 1.

**Table 1:** Patient characteristics (*n* = 150)

		Number
Sex	Male/female	106/44
Age (y)	Median (range)	61 (16–78)
Tumor site^a^	U/M/L/other	47/61/34/8
ECOG PS	0/1/2	128/21/1
Stage	I/II/III/IV	1/6/12/131
Lauren classification^b^	intestinal/diffuse/mixed/unknown	37/100/12/1
Primary tumor resection	Yes/no	82/68
Liver metastasis	Yes/no	46/104
Lymph node metastasis	Yes/no	94/56
Peritoneal metastasis	Yes/no	70/80
Number of metastatic sites	≤ 2/> 2	127/23
Second-line chemotherapy	Yes/No	104/46

a Tumor site was classified based on the Japanese Classification of Gastric Carcinoma (3rd English edition): U, upper third; M, middle third; L, lower third.

b For Lauren classification, pap, tub, and por1 with type 1 or 2 were defined as intestinal type, and others as diffuse type.

### Detection of KRAS mutations using direct sequencing

*KRAS* mutation was detected in six (4.2%) of 144 patients: at codon 12 in five (3.5%) and codon 13 in one (0.7%). *KRAS* codon 12 mutation consisted of G12D and codon 13 mutation consisted of G13D.

### Detection of gene amplification using PCR

*FGFR2*, *EGFR*, *HER2*, *MET* and *KRAS* gene amplification was suggested in 4.4%, 5.9%, 9%, 3.7% and 10.3% of patients, respectively. In diffuse-type GC, *FGFR2*, *EGFR*, *HER2*, *MET* and *KRAS* gene amplification was suggested in 5.9%, 5.9%, 5.9%, 3.0% and 9.8% of patients, respectively. In intestinal-type GC, *FGFR2*, *EGFR*, *HER2*, *MET* and *KRAS* gene amplification was suggested in 0%, 5.7%, 11.4%, 8.6% and 8.6% of patients, respectively. The frequency of overlapped amplifications among *FGFR2*, *KRAS*, *EGFR*, *HER2* and *MET* was observed in eight cases. Three (2.1%) of 144 tumors exhibited high level amplification of one component, with low level amplification of another (high *HER2*/low *MET* amplification, high *HER2*/low *KRAS* amplification, and high *EGFR*/low *KRAS* amplification in one case each). Four (2.8%) of 144 tumors exhibited a similar level of amplification between them (*EGFR*/amplification in three cases and *HER2*/*FGFR2* amplification in one case). One tumor exhibited high-level amplification of a component with two low-level amplifications (high *FGFR2*/low *KRAS* and *EGFR* amplification in one case). No tumors exhibited high-level amplification of two RTK/RAS components.

### Immunohistochemistry

The distribution of FGFR2 and MET expression was observed diffusely in tumors (Supplementary Figure 1A, B). FGFR2 membranous (FGFR2-m) overexpression (more than median H score) was observed in 55% of tumors. FGFR2 cytoplasmic (FGFR2-c) overexpression was observed in 47% of tumors. Similarly, MET membranous (MET-m) overexpression was observed in 51% of tumors. MET cytoplasmic (MET-c) overexpression was observed in 49% of tumors. The distribution of EGFR and HER2 expression was observed locally in tumors (Supplementary Figure 1C, D). EGFR overexpression was observed in 25% of tumors and EGFR membranous-positive staining (EGFR positive) in 10%.

### Correlation between gene copy and protein expression

Table 2 shows the correlation coefficient of pair-wise between each gene copy number and protein expression. There was close correlation between gene copy number and protein expression in EGFR and HER2 but not MET.

**Table 2:** Correlation coefficient of pairwise between each gene copy number and protein expression

DNA copy number	IHC-score	correlation coefficient (95% CI)	*p* value
HER2	HER2	0.751 (0.667-0.8162)	<0.001
FGFR2	FGFR2-m	0.725 (0.634-0.786)	<0.001
EGFR	EGFR-m	0.642 (0.531-0.731)	<0.001
HER2	HER2	0.488 (0.348-0.607)	<0.001
FGFR2	FGFR-c	0.23 (0.064-0.383)	0.007
MET	cMET-c	0.157 (−0.012-0.317)	0.069
MET	cMET-m	0.118 (−0.052-0.281)	0.172

FGFR2-m; FGFR2 membranous overexpression, EGFR-m; EGFR membranous overexpression, FGFR2-c; FGFR2 cytoplasmic overexpression, MET-c; MET cytoplasmic overexpression, MET-m; MET membranous overexpression.

### Relationship with overall survival (OS)

**Figure 1:**
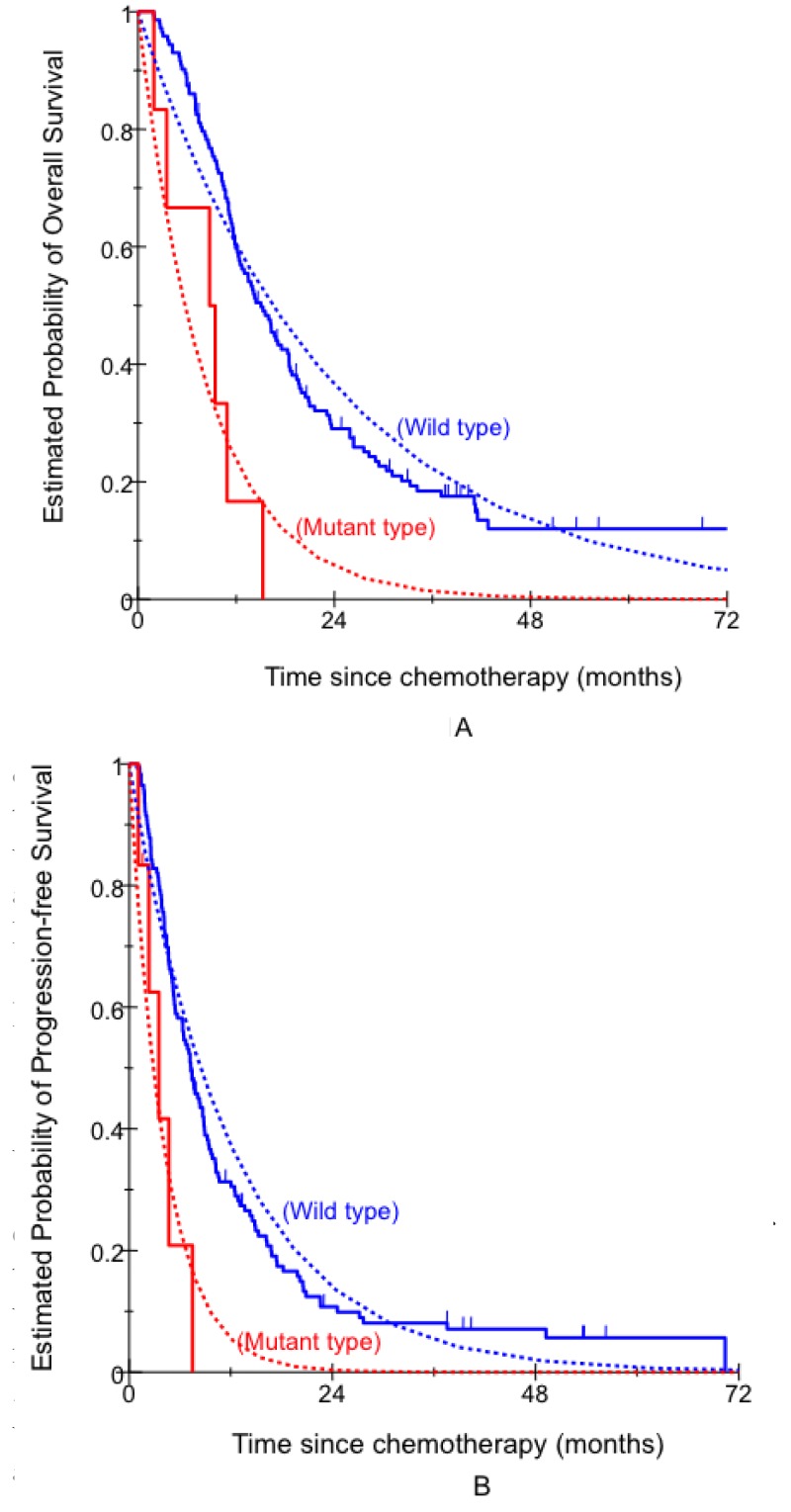
Comparison of clinical outcome by KRAS status A. OS; B. PFS.

**Figure 2:**
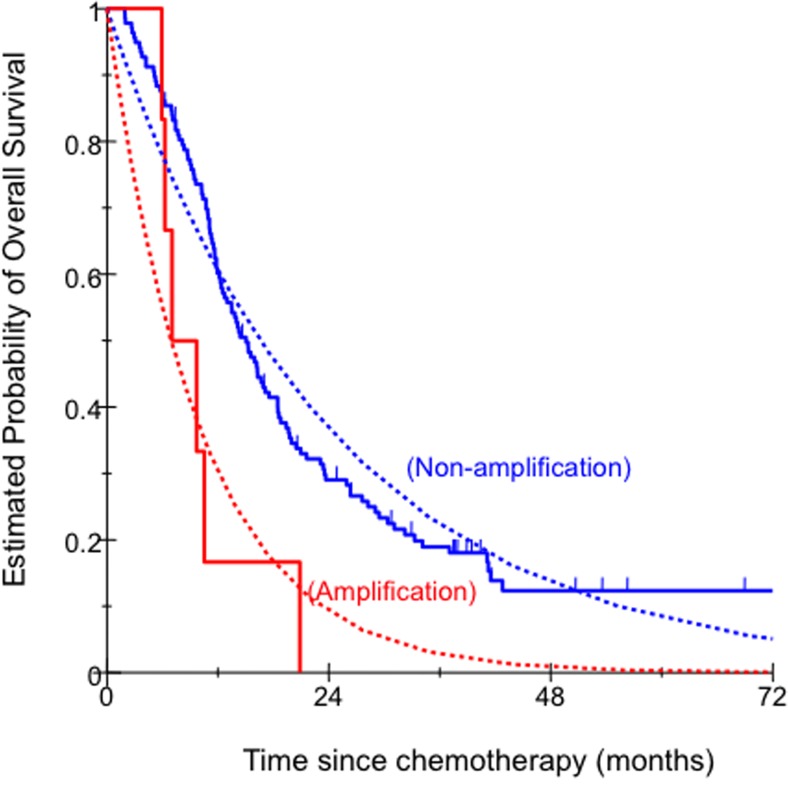
Comparison of OS by *MET* copy number

In univariate analysis, *KRAS* status indicated that *KRAS* mutant type was associated with significantly shorter OS. Patients with *KRAS* mutant type had significantly shorter median OS compared with those with *KRAS* wild type (Figure 1A, Table 3). In univariate analysis, *MET* amplification was associated with significantly shorter OS. Patients with *MET* amplification had significantly shorter median OS compared with those with *MET* non-amplification (Figure 2, Table 2). In multivariate analysis, *KRAS* mutant type and *MET* amplification remained significantly associated with OS (Table 3). No significant associations were seen for the other factors (Supplementary Table 1).

**Table 3:** Association between RTK/RAS-related genes and clinical outcome

		OS			PFS		
	N	Median, months (95%CI)	HR (95%CI)^a^	HR (95%CI)^b^	Median, (95%CI)	HR (95%CI)^a^	HR (95%CI)^b^
KRAS							
Wild type	144	14.9 (12.3–18.3)	1 (Reference)	1 (Reference)	7.3 (5.5–8.8)	1 (Reference)	1 (Reference)
Mutant type	6	8.8 (2.0–10.9)	10.97 (2.5–48.26)	5.06 (1.96–13.08)	3.1 (1.1–7.5)	8.66 (1.81–41.4)	5.93 (1.9–18.51)
***p*** value			0.005	< 0.001		0.021	0.002
MET							
Non-amp	138	14.9 (12.3–17.5)	1 (Reference)	1 (Reference)	7.3 (5.7–8.8)	1 (Reference)	1 (Reference)
Amp	6	7.0 (5.9–10.5)	5.2 (1.40–19.27)	4.81 (1.53–15.12)	4.7 (0–20.5)	1.55 (0.54–4.51)	1.83(0.57–5.81)
p value			0.033	0.007		0.59	0.3

Non-amp, non-amplification; Amp, amplification.

P value was based on log-rank test for PFS and OS in the univariate analysis (^a^) and Wald test for PFS and OS in the multivariable Cox regression model adjusting for age, Eastern Cooperative Oncology Group performance status, primary tumor site, number of metastatic sites, and liver involvement (^b^).

### Relationship with progression-free survival (PFS)

In univariate analysis, patients with *KRAS* mutant type had a significantly shorter median PFS compared with those with *KRAS* wild type (Figure 1B, Table 3). In multivariate analysis, *KRAS* mutant type remained significantly associated with PFS (Table 3). No significant associations were seen for the other factors (Supplementary Table 1).

## DISCUSSION

To the best of our knowledge, we are the first to demonstrate that *KRAS* mutation in tumors independently predicts worse PFS and OS in patients with metastatic and recurrent GC treated with SP. Yang *et al*. [17] reported that *KRAS* mutation activates *NF-κB* to promote cisplatin-resistant cancer cells. No targeted therapies for *KRAS* mutant cancers are approved, because KRAS itself has proven difficult to target directly with small molecules. MEK inhibitors are the most effective agents in *KRAS* mutant cancer cell lines [18, 19]. It is reported that GC cell lines, which have *MEK1* mutation or *MEK1* and *KRAS* mutations, are hypersensitive to MEK1 inhibitors [20]. However, MEK inhibitors have failed to induce clinical responses in *KRAS* mutant advanced non-small cell lung cancer [21, 22]. That is because KRAS activates multiple critical effectors, such as the MEK–extracellular signal-regulated kinase, phosphoinositide 3-kinase (PI3K)–AKT, and nuclear factor-κB pathways. Preclinical studies have demonstrated that combining MEK inhibitors with PI3K inhibitors or BCL-XL may be an effective therapeutic strategy for *KRAS* mutant lung cancer [23, 24]. Monotherapy with MEK1 inhibitor and combination therapy with MEK1 and PI3K inhibitors or BCL-XL might be new effective strategies for *KRAS* mutant GC.

Our study indicated that *KRAS* mutation, but not *KRAS* amplification, was associated with PFS and OS in patients with metastatic and recurrent GC treated with SP without anti-EGFR antibody. *KRAS* mutations are known to be infrequent molecular events in esophagogastric adenocarcinoma [25]. The 4.1% frequency of *KRAS* mutation in our population is in keeping with the 3–10% reported in other studies [26, 27]. *KRAS* mutants were not detected in five RTK-related gene amplifications in our population. Anti-EGFR antibody was expected to be effective against GC, because *KRAS* mutations are infrequent molecular events [28,29], and *KRAS*-mutated GC was not affected by capeciatbine+cisplatin with or without cetuximab in our study. However, current clinical studies have reported that addition of EGFR antibodies to chemotherapy did not yield additional benefit for patients with advanced gastric and gastroesophageal junction cancer. An increased *EGFR* copy number is associated with a favorable response to anti-EGFR therapy in patients with *KRAS* wild-type colorectal cancer [30-32]. We found amplification of the *EGFR* gene in nine (6.3%) of the 144 samples analyzed. *EGFR* amplification overlapped *KRAS* amplification in five (56%) of nine patients in our study. Amplification of the *KRAS* gene was identified in 13 (9%) of the 144 samples analyzed. Mita *et al*. reported that *KRAS* amplification resulted in KRAS activation in the absence of mutation [33]. Mekenkamp *et al*. reported that copy number gains in wild-type *KRAS* were associated with poor PFS in patients with metastatic colorectal cancer treated with cetuximab [34]. High-frequency amplification overlapped *EGFR* and *KRAS* and may explain why addition of anti-EGFR antibodies to chemotherapy is not beneficial in patients with GC.

Our study showed that overlaps were observed in amplifications of *FGFR2*, *KRAS*, *EGFR*, *HER2* and *MET* in eight cases. Two of the eight cases had *FGFR2* amplification. One tumor exhibited a high level of *FGFR2* amplification with a low level of *EGFR* and *KRAS* amplification. The other tumor exhibited a similar level of amplification of *FGFR2* and *HER2*. Das *et al*. reported that correlation between FGFR2 and ERBB2 expression is exclusive using multicolor FISH [35]. One limitation of our study was that we did not confirm whether overlapped *FGFR2* and *HER2* amplification in the tumor was exclusive, because we did not perform multicolor FISH. We merged between FGFR2 and HER2 overexpression, because there was close correlation between FGFR2 copy number and membranous expression, such as HER2. Each positive tumor cells between FGFR2-overexpression and HER2-overexpression were mutually exclusive (Supplementary Figure 2).

Our study has the limitation because the real-time PCR in this study cannot be said that the heterogeneity of RTK-amplification in an equivalence cell. We need to perform highly sensitive Real-time DNA Clamp PCR on extracted genomic DNA in LCM or multicolor FISH.

FISH has been widely used for detecting DNA copy number. However, small samples are limited for measurement of multiple genes using FISH. We used real-time PCR to detect DNA copy number. We identified 5 as the threshold copy number after considering that identified by FISH analysis, ≥4, with 1 as the margin value. Frequency of *FGFR2* amplification at this threshold was similar to that in other studies. We performed dual color *in situ* hybridization (DISH) in some samples to confirm whether the results of PCR were similar to those of DISH. DNA copy number analysis by PCR yielded results that were highly consistent with DISH (Supplementary Table 2). The different results for PCR and DISH may have been due to a genome domain counting the copy number of each other. HER2 positivity (immunohistochemistry score 3+ and/or DNA amplification) was 18%, which was identical to previous studies that reported that HER2 positivity (immunohistochemistry score 3+ and/or FISH-positive) in Japanese patients with GC was 10–20%.

We searched the frequency of *HER2* mutations in GC using the Cosmic database (Sanger Institute, http://cancer.sanger.ac.uk/cosmic) on November 12, 2015, and the frequency was 2.83% (25 of 884 cases). This was similar to the frequency of *HER2* mutations in colon cancer. In addition, the frequency became lower when we narrowed it down to the kinase domain of *HER2*. Therefore, it was difficult to do the analysis with a sufficient number of cases because there was a lower frequency of *HER2* than *KRAS* (codon12, 13) mutations.

Patients with *MET* amplification had significantly worse survival in our study. Some reports show that MET overexpression and higher *MET* copy number are associated with poor prognosis in patients with GC [36,37]. Fuse *et al*. reported that patients with MET overexpression had significantly worse OS than those without overexpression [38]. However, in view of the EXPAND study [7] and recent data on MET inhibitors presented at the ASCO 2015 Meeting, these results should be toned down.

In conclusion, our data show for the first time that *KRAS* mutation and *MET* amplification are promising prognostic markers in patients with metastatic and recurrent GC. Furthermore, *KRAS* mutation may be useful as a predictive marker in patients treated with SP combination chemotherapy. As this was a retrospective analysis with limited sample size, larger prospective studies are needed to validate our findings.

## MATERIALS AND METHODS

### Patients and study design

We enrolled 150 patients who had a histologically confirmed diagnosis of primary gastric adenocarcinoma and were treated with first-line SP from the Cancer Institute Hospital of the Japanese Foundation for Cancer Research (Tokyo, Japan).

Concurrent chemotherapy consisted of infusion of cisplatin (60 mg/m^2^) on days 1 and 8 plus oral S-1 (80 mg/m^2^/day) on days 1–21. This regimen was given unless there was evidence of progression, intolerable toxicity, or early withdrawal. Response was measured by computed tomography every 8 weeks, according to the Response Evaluation Criteria in Solid Tumors (RECIST) 1.0. This study was approved by the Central Ethics Committee of the Japanese Foundation for Cancer Research and the Institutional Review Boards of the Cancer Institute Hospital, and all patients gave signed informed consent for the analysis of molecular correlates.

### Detection of KRAS mutation

For *KRAS* mutation detection in formalin-fixed paraffin-embedded (FFPE) samples, we used a PCR followed by direct sequencing. Genomic DNA was extracted from FFPE samples using the QIAamp DNA FFPE Tissue Kit (Qiagen, Tokyo, Japan). RNase A (Qiagen) was used to digest single-strand RNA for the isolation of RNA-free DNA. DNA was quantified first by UV absorption (NanoDrop 2000 UV-Vis Spectrophotometer; Thermo Fisher Scientific, Kanagawa, Japan). The amplification products were analyzed in an ABI 3130xl Genetic Analyzer (Life Technologies Japan; Applied Biosystems, Tokyo, Japan).

### Detection of gene amplification using real-time PCR

Starting DNA material was the same as that for *KRAS* mutation detection. Real-time PCR was performed using the TaqMan probes in an ABI 7900HT Real-Time PCR instrument (ABI, Foster City, CA, USA). Commercially available FAM-dye-labeled probes were designed to amplify *HER2*, *EGFR*, *FGFR2*, *MET* and *KRAS*. VIC-dye-labeled ribonuclease P RNA component H1 (RPPH1), telomerase reverse transcriptase (TERT) and human genomic DNA (Promega, Madison, WI, USA) were used as the endogenous control probes and control sample because *RPPH1* and *TERT* have exactly two copies per diploid human genome in healthy tissue, which are located on chromosome 14q11.2 and 5p15.33, respectively. The TaqMan copy number assay contained 1 μl *HER2*, *EGFR*, *FGFR2*, *MET* and *KRAS* probe (20×, FAM labeled), 1 μl *RPPH1* or *TERT* probe mix (20×, VIC labeled), 10 μl TaqMan Universal PCR Master Mix (2×), 1.5 μl genomic DNA and 6.5 μl water. PCR cycling conditions were 95°C for 10 min, followed by two-step cycling: 40 cycles of 95°C for 15 s and 60°C for 1 min. A manual threshold cycle threshold (Ct) of 0.2 and an automatic baseline were used to detect the template quantity of target genes and *RPPH1* or *TERT* using ABI sequence detection software version 2.4. The target probes and internal control were loaded in the same well and each reaction was performed in quadruplicate. CopyCaller version 2.0 (ABI) was used to calculate the copy number of each probe based on the real-time PCR data. Copy number of each target gene was calculated as the average of those based on two reference genes. The threshold of each gene amplification was identified as ≥5.

### Immunohistochemistry

FFPE blocks were cut into 4-μm serial sections. Immunohistochemical staining for HER2, EGFR, FGFR2 and MET was performed with Histofine Histostainer 36A (Nichirei Biosciences, Tokyo, Japan) using primary antibodies against HER2 (I-View Pathway anti-HER2/neu 4B5; Ventana), EGFR (Confirm anti-EGFR 3C6; Ventana), FGFR2 (K-sam rabbit IgG; IBL, Gunma, Japan; 1: 20) and MET (anti-c-Met rabbit polyclonal antibody; Invitrogen, Carlsbad, CA, USA; 1: 200). The sections were deparaffinized in xylene and dehydrated with graded ethanol. After being washed in tap water, the sections were placed in retrieval buffer (Cell Conditioning 1; Ventana). For antigen retrieval, the sections were heated in a water bath at 90°C for 30 min for HER2 and EGFR staining, and heated in a Pascal Pressure Chamber (Dako, Tokyo, Japan) at 125°C for 30 s and 90°C for 10 s for FGFR2 and MET staining. After washing with PBS, the sections were immersed in peroxidase blocking solution (Dako) to block endogenous peroxidase activity at room temperature for 10 min. Individual section slides were then incubated with primary antibodies at room temperature for 1 h. After further washing with PBS, the sections were incubated with the following secondary antibodies: NIEW Biotinylated Ig Secondary Antibody (Ventana) for HER2 and EGFR, and N-Histofine Simple Stain MAX PO (R) for FGFR2 and MET. After incubating with streptavidin horseradish peroxidase (NIEW SA-HRP; Ventana) at room temperature for 30 min, except for FGFR2 and MET, the sections were washed with PBS, and were subsequently incubated with 3,3′-diaminobenzidine tetrahydrochloride (Dako) at room temperature for 3 min. The sections were then counterstained with Dako REAL Hematoxylin.

### Immunohistochemical analysis

Immunostaining was assessed and scored subjectively by two pathologists blinded to clinical characteristics and outcomes. For HER2, EGFR, MET and FGFR2, immunostaining of the tumor cell membrane was scored using a four-grade scale (0, 1+, 2+ and 3+) and area percentage of positive cells (0–100%) in each grade according to scoring in GC. Immunostaining of the tumor cell cytoplasm was scored using a four-grade scale (0, 1+, 2+ and 3+) and area percentage of positive cells in each grade. MET and FGFR2 reactivity was scored as 0 if there was no cytoplasmic reactivity within the tumor, or as 1+, 2+ or 3+ depending on the intensity above the background level. For HER2, EGFR, FGFR2 and MET, we calculated H score by multiplying the staining intensity [0 (grade 0), 1 (grade 1+), 2 (grade 2+), 3 (grade 3+)] with the percentage of positive cells at each grade, and all values were added to obtain the final immunohistochemistry score, ranging from 0 to 300. HER2, EGFR and MET were scored using a four-grade scale (0, 1+, 2+ and 3+) according to Hofmann's criteria [38]. FGFR2 was scored using a four-grade scale (grade 0, staining reactivity in <50% of cancer cells; grade 1+, cytoplasm and/or nuclear reactivity with an intensity score of 1+ in >50% of cancer cells, grade 2+, cytoplasm and/or nuclear reactivity with an intensity score of 2+ in >50% of cancer cells; grade 3+, cytoplasm and/or nuclear reactivity with an intensity score of 3+ in >50% of cancer cells) according to the criteria by Nagatsuma *et al*. [39]. The other evaluation of HER2, FGFR2 and MET was performed according to the defined negativity (grade 0 and 1+) and positivity (grade 2+ and 3+). EGFR was evaluated according to the defined negativity (grade 0, 1+ and 2+) and positivity (grade 3+).

### Statistical analysis

The primary endpoint of this retrospective study was OS, and the secondary endpoint was PFS. OS was defined as the period from starting therapy to the date of death or censoring of the last contact if alive. PFS was defined as the interval from the first day of starting first-line SP therapy to the first day of documented disease progression or death. If progression or death was not observed, PFS was censored on the day of the last computed tomography scan. All factors were tested for deviation from Hardy–Weinberg equilibrium using the χ^2^ test with 1 degree of freedom.

The associations between all factors and OS and PFS were examined in univariate analysis using Kaplan–Meier estimation and Cox–Mantel tests, and in multivariate analysis using a Cox proportional hazards regression model. The following baseline characteristics were adjusted in the multivariate analysis: sex, age, Eastern Cooperative Oncology Group performance status, Lauren classification, tumor location, primary tumor resection, lung metastases, liver metastases, peritoneal metastases, lymph node metastases, and ≥2 metastases. The relationships between all factors and response were assessed in the univariate analysis using χ^2^ tests and in the multivariate analysis using a logistic regression model controlling for potential predictive variables. *P* values for all factors were adjusted for multiple testing using a modified test of Conneely and Boehnke that was applied for the correlated tests owing to linkage disequilibrium and the different modes of inheritance considered.

R version 3.2.0 software (http://www.r-project.org) was used to conduct all the analyses by Sugimoto Data Analysis Service (Aichi, Japan). All tests were two-sided at a significance level of 0.05.

## SUPPLEMENTARY MATERIAL FIGURES AND TABLES



## References

[1] Koizumi W, Narahara H, Hara T, Takagane A, Akiya T, Takagi M, Miyashita K, Nishizaki T, Kobayashi O, Takiyama W, Toh Y, Nagaie T, Takagi S (2008). S-1 plus cisplatin versus S-1 alone for first-line treatment of advanced gastric cancer (SPIRITS trial): a phase III trial. Lancet Oncol.

[2] Price TJ, Shapiro JD, Segelov E, Karapetis CS, Pavlakis N, Van Cutsem E, Shah MA, Kang YK, Tebbutt NC (2012). Management of advanced gastric cancer. Expert Rev Gastroenterol Hepatol.

[3] Deng N, Goh LK, Wang H, Das K, Tao J, Tan IB, Zhang S, Lee M, Wu J, Lim KH, Lei Z, Goh G, Lim QY (2012). A comprehensive survey of genomic alteration in gastric cancer reveals systematic patterns of molecular exclusivity and co-occurrence among distinct therapeutic targets. Gut.

[4] Cuello M, Ettenberg SA, Clark AS, Keane MM, Posner RH, Nau MM, Dennis PA, Lipkowitz S (2001). Down-regulation of the erbB-2 receptor by trastuzumab enhances tumor necrosis factor related apoptosis inducing ligand mediated apoptosis in breast and ovarian cancer cell lines that overexpress erbB-2. Cancer Res.

[5] Bang YJ, Van Cutsem E, Feyereislova A, Chung HC, Shen L, Sawaki A, Lordick F, Ohtsu A, Omuro Y, Satoh T, Aprile G, Kulikov E, Hill J (2010). Trastuzumab in combination with chemotherapy versus chemotherapy alone for treatment of HER2-positive advanced gastric or gastro-oesophageal junction cancer (ToGA): a phase 3, open-label, randomized controlled trial. Lancet.

[6] Kim MA, Lee HS, Lee HE, Jeon YK, Yang HK, Kim WH (2008). EGFR in gastric carcinoma: prognostic significance of protein overexpression and high gene copy number. Histopathology.

[7] Lordick F, Kang YK, Chung HC, Salman P, Oh SC, Bodoky G, Kurteva G, Volovat C, Moiseyenko VM, Gorbunova V, Park JO, Sawaki A, Celik I (2013). Capecitabine and cisplatin with or without cetuximab for patients with previously untreated advanced gastric cancer (EXPAND): a randomized, open-label phase 3 trial. Lancet Oncol.

[8] Waddell T, Chau I, Cunningham D, Gonzalez D, Okines AF, Okines C, Wotherspoon A, Saffery C, Middleton G, Wadsley J, Ferry D, Mansoor W, Crosby T (2013). Epirubicin, oxaliplatin, and capecitabine with or without panitumumab for patients with previously untreated advanced oesophagogastric cancer (REAL3): a randomized, open-label phase 3 trial. Lancet Oncol.

[9] Gheerardi E, Birchmeier W, Birchmeier C, Vande Woude G (2012). Targeting MET in cancer: rationale and progress. Nat Rev Cancer.

[10] Huang TJ, Wang JY, Lin SR, Lian ST, Hsieh JS (2001). Overexpression of c-met protooncogene in human gastric carcinoma-correlation to clinical features. Acta Oncol.

[11] Toiyama Y, Yasuda H, Saigusa S, Matushita K, Fujikawa H, Tanaka K, Mohri Y, Inoue Y, Goel A, Kusunoki M (2012). Co-expression of hepatocyte growth factor and c-Met predicts peritoneal dissemination established by autocrine hepatocyte growth factor/c-Met signaling in gastric cancer. Int J Cancer.

[12] Lee J, Seo JW, Jun HJ, Ki CS, Park SH, Park YS, Lim HY, Choi MG, Bae JM, Sohn TS, Noh JH, Kim S, Jang HL (2011). Impact of MET amplification on gastric cancer: possible roles as a novel prognostic marker and a potential therapeutic target. Oncol Rep.

[13] Iveson T, Donehower RC, Davidenko I, Tjulandin S, Deptala A, Harrison M, Nirni S, Lakshmaiah K, Thomas A, Jiang Y, Zhu M, Tang R, Anderson A (2014). Rilotumumab in combination with epirubicin, cisplatin, and capecitabine as first-line treatment for gastric or oesophagogastric junction adenocarcinoma: an open-label, dose de-escalation phase 1b study and a double-blind, randomized phase 2 study. Lancet Oncol.

[14] Doshi S, Gisleskog PO, Zhang Y, Zhu M, Oliner KS, Loh E, Ruixo JJ (2015). Rilotumumab exposure-response relationship in patients with advanced or metastatic gastric cancer. Clin Cancer Res.

[15] Bai A, Meetze K, Vo NY, Kollipara S, Mazsa EK, Winston WM, Weiler S, Poling LL, Chen T, Ismail NS, Jiang J, Lerner L, Gyuris J (2010). GP369, an FGFR2-IIIb-specific antibody, exhibits potent antitumor activity against human cancers driven by activated FGFR2 signaling. Cancer Res.

[16] Su X, Zhan P, Gavine PR, Morgan S, Womack C, Ni X, Ni X, Shen D, Bang YJ, Im SA, Ho Kim W, Jung EJ, Grabsch HI, Kilgour E (2014). FGFR2 amplification has prognostic significance in gastric cancer: results from a large international multicenter study. Br J Cancer.

[17] Yang L, Zhou Y, Li Y, Zhou J, Wu Y, Cui Y, Yang G, Hong Y (2015). Mutations of p53 and KRAS activate NF-kB to promote chemoresistance and tumorigenesis via dysregulation of cell cycle and suppression of apoptosis in lung cancer cells. Cancer Lett.

[18] Jing J, Greshock J, Holbrrok JD, Gilmartin A, Zhang X, McNeil E, Conway T, Moy C, Laquerre S, Bachman K, Wooster R, Degenhardt Y (2012). Comprehensive predictive biomarker analysis for MEK inhibitor GSK 1120212. Mol Cancer Ther.

[19] Liu L, Shi H, Zhang V, Gilmer T (2011). Identification of molecular determinants of response to GSK1120212B, a potent and selective MEK inhibitor, as a single agent and in combination in RAS/RAF mutant non small cell lung carcinoma cells. Cancer Res.

[20] Sogabe S, Togashi Y, Kato H, Kogita A, Mizukami T, Sakamoto Y, Banno E, Terashima M, Hayashi H, de Velasco MA, Sakai K, Fujita Y, Tomida S (2014). MEK inhibitor for gastric cancer with MEK1 gene mutations. Mol Cancer Ther.

[21] Carter CA, Rajan A, Szabo E, Khozin S, Thomas A, Brzezniak CE, Guha U, Austin Doyle L, Keen C, Steinberg SM, Xi L, Raffeld M, Reckamp KL (2013). Two parallel randomized phase II studies of selumetinib (S) and erlotinib (E) in advanced non-small cell lung cancer selected by KRAS mutations. J Clin Oncol.

[22] Blumenschein G, Smit EF, Planchard D, Kim DW, Cadranel J, De Pas T, Dunphy F, Udud K, Ahn MJ, Hanna NH, Kim JH, Mazieres J, Kim SW (2015). A randomized phase 2 of the MEK1/MEK2 inhibitor trametinib (GSK1120212) compared with docetaxel in KRAS-mutant advanced non-small cell lung cancer (NSCLC). Ann Oncol.

[23] Engelman JA, Chen L, Tan X, Crosby K, Guimaraes AR, Upadhyay R, Maria M, McNamara K, Perera SA, Song Y, Chirieac LR, Kaur R, Lightbown A (2008). Effective use of PI3K and MEK inhibitors to treat mutant Kras G12D and PIK3CA H1047R murine lung cancers. Nat Med.

[24] Corcoran RB, Cheng KA, Hata AN, Faber AC, Ebi H, Coffee EM, Greninger P, Brown RD, Godfrey JT, Cohoon TJ, Song Y, Lifshits E, Hung KE (2013). Synthetic lethal interaction of combined BCL-XL and MEK inhibition promotes tumor regressions in KRAS mutant cancer models. Cancer Cell.

[25] Kim IJ, Park JH, Kang HC, Shin Y, Park HW, Park HR, Ku JL, Lim SB, Park JG (2003). Mutational analysis of BRAF and K-ras in gastric cancers: absence of BRAF mutations in gastric cancers. Hum Genet.

[26] Moehler M, Mueller A, Trarbach T, Lordick F, Seufferlein T, Kubicka S, Geissler M, Schwarz S, Galle PR (2011). Cetuximab with irinotecan folinic acid and 5-fluorouracil as first-line treatment in advanced gastroesophageal cancer: a prospective multi-center biomarker-oriented phase II study. Ann Oncol.

[27] Lordick F, Luber B, Lorenzen S, Hegewisch-Becker S, Folprecht G, Wöll E, Decker T, Endlicher E, Rothling N, Schuster T, Keller G, Fend F, Peschel C (2010). Cetuximab plus oxaliplatin/leucovorin/5-fluorouracil in first-line metastatic gastric cancer: a phase II study of the Arbeitsgemeinschaft Internistische Onkologie (AIO). Br J Cancer.

[28] Moutinho C, Mateus AR, Milanezi F, Carneiro F, Seruca R, Suriano G (2008). Epidermal growth factor receptor structural alterations in gastric cancer. BMC Cancer.

[29] Tsugawa K, Yonemura Y, Hirono Y, Fushida S, Kaji M, Miwa K, Miyazaki I, Yamamoto H (1998). Amplification of c-met, c-erbB-2 and epidermal growth factor receptor gene in human gastric cancers: correlation to clinical features. Oncology.

[30] Jiang Z, Li C, Li F, Wang X (2013). EGFR gene copy number as a prognostic marker in colorectal cancer patients treated with cetuximab or panitumumab: a systematic review and meta analysis. PloS One.

[31] Cesare MD, Lauricella C, Veronese SM, Cominetti D, Pisano C, Zaffaroni N, Zuco V (2014). Synergistic antitumor activity of cetuximab and namitecan in human squamous cell carcinoma model relies on cooperative inhibition of EGFR expression and depends on high EGFR gene copy number. Clin Cancer Res.

[32] Moroni M, Veronese S, Benvenuti S, Marrapese G, Sartore-Bianchi A, Di Nicolantonio F, Gambacorta M, Siena S, Bardelli A (2005). Gene copy number for epidermal growth factor receptor (EGFR) and clinical response to antiEGFR treatment in colorectal cancer: s cohort study. Lancer Oncol.

[33] Mita H, Toyota M, Aoki F, Akashi H, Maruyama R, Sasaki Y, Suzuki H, Idogawa M, Kashima L, Yanagihara K, Fujita M, Hosokawa M, Kusano M (2009). A novel method, digital genome scanning detects KRAS gene amplification in gastric cancers: involvement of overexpressed wild-type KRAS in downstream signaling and cancer cell growth. BMC Cancer.

[34] Mekenkamp LJM, Tol J, Dijkstra JR, de Krijger I, Vink-Börger ME, van Vliet S, Teerenstra S, Kamping E, Verwiel E, Koopman M, Meijer GA, van Krieken JH, Kuiper R (2012). Beyond KRAS mutation status: influence of KRAS copy number status and microRNAs on clinical outcome to cetuximab in metastatic colorectal cancer patients. BMC Cancer.

[35] Das K, Gunasegaran B, Tan IB (2014). Mutually exclusive FGFR2, HER2, and KRAS gene amplifications in gastric cancer revealed by multicolour FISH. Cancer Lett.

[36] Nakajima M, Sawada H, Yamada Y, Watanabe A, Tatsumi M, Yamashita J, Matsuda M, Sakaguchi T, Hirao T, Nakano H (1999). The prognostic significance of amplification and overexpression of c-met and c-erbB-2 in human gastric carcinomas. Cancer.

[37] Yu S, Yu Y, Zhao N, Cui J, Li W, Liu T (2013). c-Met as a prognostic marker in gastric cancer: a systematic review and meta-analysis. PLoS One.

[38] Fuse N, Kuboki Y, Kuwata T, Nishina T, Kadowaki S, Shinozaki E, Machida N, Yuki S, Ooki A, Kajiura S, Kimura T, Yamanaka T, Shitara K (2015). Prognostic impact of HER2, EGFR, and c-MET status on overall survival of advanced gastric cancer patients. Gastric Cancer.

