# Supplementary material for: Prognostic impact of KRAS mutant type and MET amplification in metastatic and recurrent gastric cancer patients treated with first-line S-1 plus cisplatin chemotherapy
Source: Genes Cancer. 2016 Jan;7(1-2):27–35. doi: 10.18632/genesandcancer.96 (PMC4773703; doi:10.18632/genesandcancer.96)
Supplement: Supplementary file 1 [file ganc-07-027-s001.pdf]

Prognostic impact of KRAS mutant type and MET amplification in metastatic and recurrent gastric cancer patients treated with first-line S-1 plus cisplatin chemotherapy

Supplementary Material

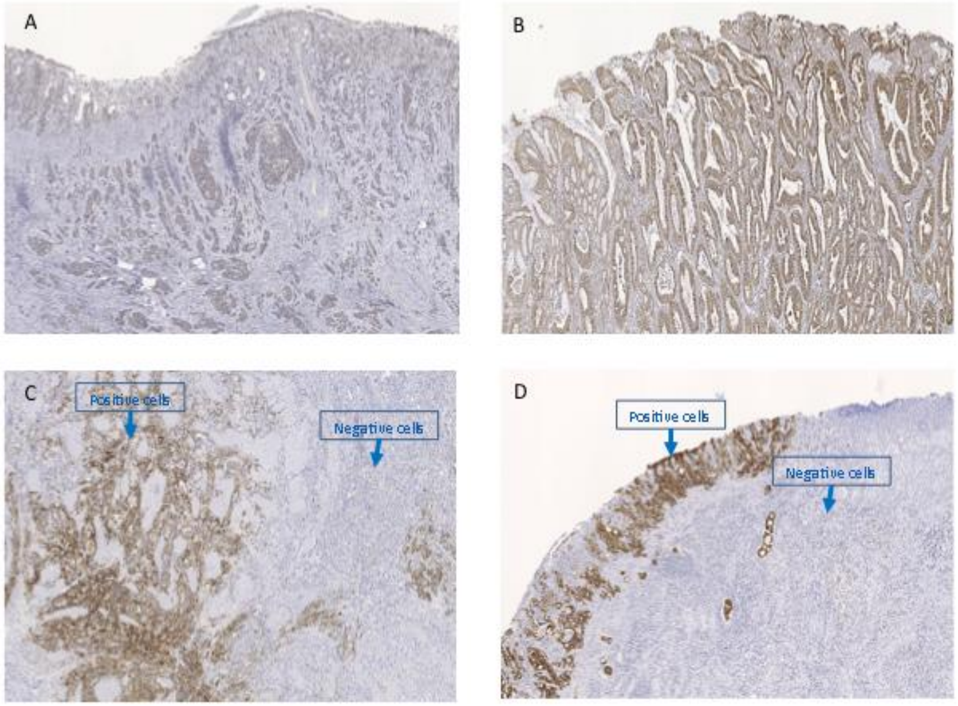

Supplementary Figure 1: Distribution of immunochemical staining in tumor. FGFR2 (**A**) and MET (**B**) diffuse distribution of positive cancer cells; EGFR (**C**) and HER2 (**D**) heterogeneous distribution of positive cancer cells.

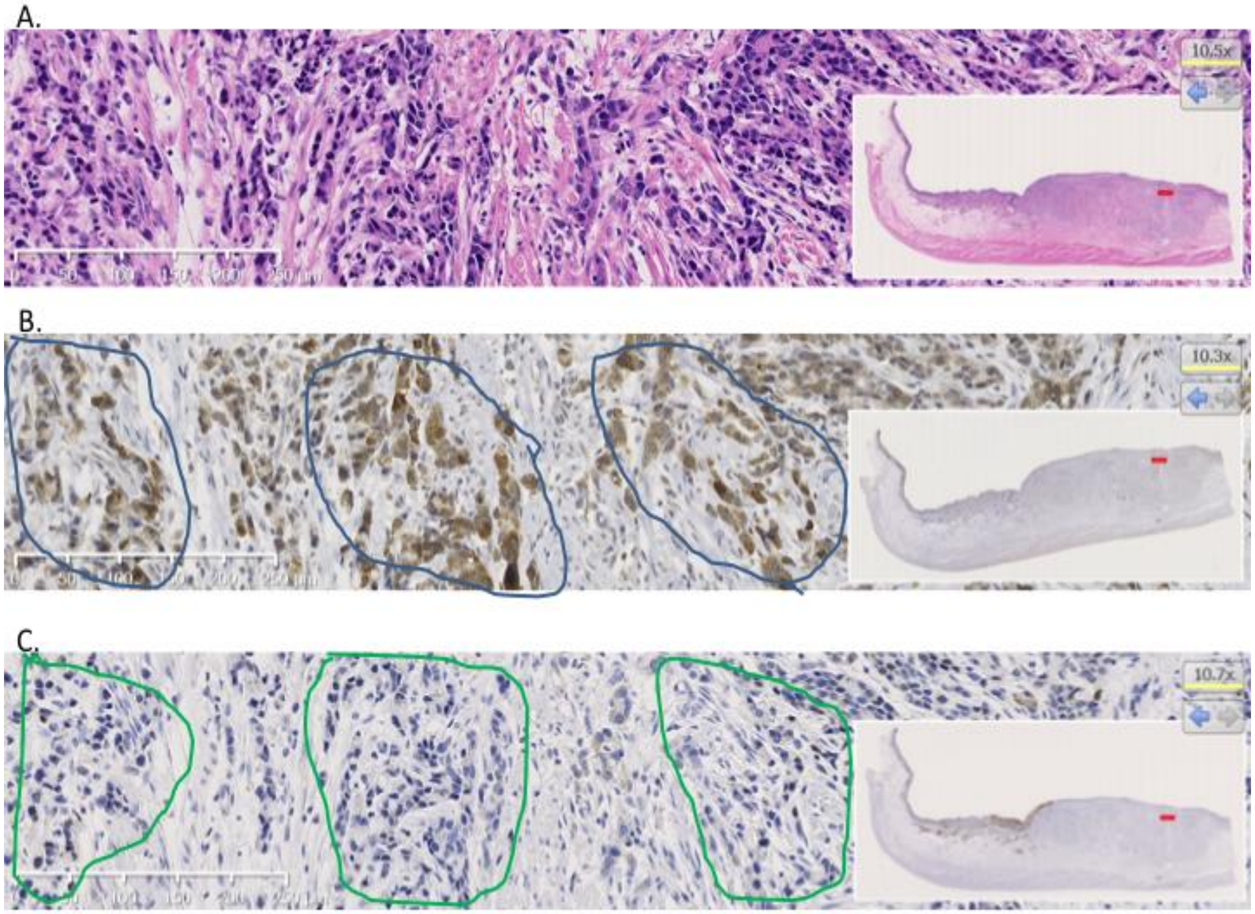

Supplementary Figure 2: Distribution of immunochemical staining in tumor. Hematoxylin and eosin staining (A); FGFR (B) and HER2 (C) heterogeneous distribution of positive cancer cells.

Supplementary Table 1. Association between RTK/RAS-related genes and clinical outcome

	OS				PFS		
	N	Median, months (95%CI)	HR (95%CI)[a]	HR (95%CI)[b]	Median, months (95%CI)	HR (95%CI)[a]	HR (95%CI)[b]
HER2							
Non amp	132	14.2 (11.9–17.1)	1 (Reference)	1 (Reference)	7.2 (5.4–8.3)	1 (Reference)	1 (Reference)
Amp	13	12.5 (7.7–29.4)	0.82 (0.45–1.5)	2.02 (0.83–4.95)	9.9 (2.6–18.2)	0.8 (0.44–1.45)	1.34 (0.55–3.37)
p value			0.63	0.12		0.56	0.53
EGFR							
Non amp	136	14.0 (11.8–16.9)	1 (Reference)	1 (Reference)	7.2 (5.4–8.3)	1 (Reference)	1 (Reference)
Amp	9	16.4 (11.1–26.3)	0.82 (0.41–1.63)	0.58 (0.19–1.76)	9.5 (2.4–27.2)	0.74 (0.38–1.42)	0.30 (0.10–0.92)
p value			0.69	0.34		0.46	0.035
FGFR2							
Non amp	138	14.3 (11.8–17.5)	1 (Reference)	1 (Reference)	7.2 (5.5–8.7)	1 (Reference)	1 (Reference)
Amp	6	9.0 (0–33.3)	1.82 (0.59–5.56)	0.75 (0.21–2.75)	4.6 (1.3–14.8)	2.06 (0.56–7.54)	1.58 (0.43-5.84)
p value			0.45	0.67		0.45	0.49
KRAS							
Non amp	132	14.0 (11.7–16.9)	1 (Reference)	1 (Reference)	7.2 (5.4–8.4)	1 (Reference)	1 (Reference)
Amp	13	17.2 (11.1–53.6)	0.71 (0.40–1.26)	1.31 (0.56–3.08)	8.1 (3.5–27.2)	0.74 (0.42–1.3)	1.67 (0.72–3.91)
p value			0.3	0.54		0.37	0.23
FGFR2-m							

≤ median value	60	12.2 (9.8–16.3)	1 (Reference)	1 (Reference)	5.7 (4.6–8.7)	1 (Reference)	1 (Reference)
> median value	75	15.3 (11.8–18.5)	0.88 (0.61–1.29)	0.94 (0.57–1.60)	7.3 (4.9–9.4)	0.97 (0.67–1.40)	0.86 (0.54–1.37)
p value			0.59	0.77		0.95	0.53
FGFR2-c							
≤ median value	73	12.7 (11.1–16.3)	1 (Reference)	1 (Reference)	7.3 (5.2–9.2)	1 (Reference)	1 (Reference)
> median value	62	15.3 (11.4–18.5)	1.0 (0.67–1.45)	0.95 (0.57–1.60)	6.3 (4.2–8.7)	1.29 (0.89–1.87)	1.30 (0.79–2.16)
p value			0.94	0.85		0.21	0.3
cMET-m							
≤ median value	64	15.8 (11.6–18.8)	1 (Reference)	1 (Reference)	8.6 (5.2–10.2)	1 (Reference)	1 (Reference)
> median value	71	12.6 (10.9–16.3)	1.27 (0.88–1.85)	1.02 (0.60–1.75)	6.3 (4.7–7.4)	1.34 (0.92–1.93)	0.99 (0.57–1.73)
p value			0.24	0.93		0.14	0.98
cMET-c							
≤ median value	70	13 (11.3–16.3)	1 (Reference)	1 (Reference)	7.0 (5.1–8.4)	1 (Reference)	1 (Reference)
> median value	65	14.8 (11.1–19.8)	0.91 (0.63–1.32)	0.73 (0.45–1.18)	7.0 (4.7–9.5)	0.98 (068–1.42)	0.86 (0.54–1.39)
p value			0.68	0.2		0.1	0.55
HER2							
≤ median value	81	13.5 (11.4–16.9)	1 (Reference)	1 (Reference)	7.1 (4.9–8.3)	1 (Reference)	1 (Reference)
> median value	54	14.8 (11.1–19.4)	1.01 (0.69–1.49)	0.90 (0.54–1.50)	6.5 (4.7–9.7)	1.09 (0.75–1.59)	0.89 (0.54–1.46)
p value			0.98	0.68		0.72	0.64
EGFR							

negative	122	13.5 (11.5–16.3)	1 (Reference)	1 (Reference)	7.2 (5.2–8.4)	1 (Reference)	1 (Reference)
positive	13	16.5 (11.1–30.4)	0.86 (0.47–1.58)	0.88 (0.36–2.20)	5.8 (3.8–12.9)	0.92 (0.50–1.68)	0.93 (0.39–2.24)
p value			0.74	0.79		0.9	0.88

Non amp, non-amplification; Amp, amplification; -m, membrane staining; -c, cell-cytoplasm staining.
P value was based on log-rank test for PFS and OS in the univariate analysis ([a]) and Wald test for PFS and OS in the multivariable Cox regression model adjusting for age, Eastern Cooperative Oncology Group performance status, primary tumor site, number of metastatic sites, and liver involvement ([b]).

Supplementary Table 2. Correlation between DNA copy number by PCR method and DISH

HER2	IHC-score	DNA copy number (PCR)	(DISH ratio)
Case 1	2	8.6	5.5
Case 2	2	1.3	1.1
Case 3	2	2.0	1.3
EGFR			
Case 4	3	42.7	21.3
Case 5	3	40.6	31.8
Case 6	3	13.62	29.7
cMET			
Case 7	3	58.9	30.1
Case 8	3	41.8	63.8
Case 9	2	19.0	14.4

Abbreviations: HER2, human epidermal growth factor receptor 2; EGFR, epithelial growth factor receptor; IHC, immunohistochemistry.
